# Rapid Detection and Quantification of Adulterants in Fruit Juices Using Machine Learning Tools and Spectroscopy Data

**DOI:** 10.3390/s22103852

**Published:** 2022-05-19

**Authors:** José Luis P. Calle, Marta Barea-Sepúlveda, Ana Ruiz-Rodríguez, José Ángel Álvarez, Marta Ferreiro-González, Miguel Palma

**Affiliations:** 1 Department of Analytical Chemistry, Faculty of Sciences, IVAGRO, CeiA3, University of Cadiz, 11510 Puerto Real, Spain; joseluis.perezcalle@uca.es (J.L.P.C.); marta.barea@uca.es (M.B.-S.); ana.ruiz@uca.es (A.R.-R.); miguel.palma@uca.es (M.P.); 2 Department of Physical Chemistry, Faculty of Sciences, INBIO, University of Cadiz, Apartado 40, 11510 Puerto Real, Spain; joseangel.alvarez@uca.es

**Keywords:** near-infrared spectroscopy, adulteration, fruits juices, machine learning, regression, classification

## Abstract

Fruit juice production is one of the most important sectors in the beverage industry, and its adulteration by adding cheaper juices is very common. This study presents a methodology based on the combination of machine learning models and near-infrared spectroscopy for the detection and quantification of juice-to-juice adulteration. We evaluated 100% squeezed apple, pineapple, and orange juices, which were adulterated with grape juice at different percentages (5%, 10%, 15%, 20%, 30%, 40%, and 50%). The spectroscopic data have been combined with different machine learning tools to develop predictive models for the control of the juice quality. The use of non-supervised techniques, specifically model-based clustering, revealed a grouping trend of the samples depending on the type of juice. The use of supervised techniques such as random forest and linear discriminant analysis models has allowed for the detection of the adulterated samples with an accuracy of 98% in the test set. In addition, a Boruta algorithm was applied which selected 89 variables as significant for adulterant quantification, and support vector regression achieved a regression coefficient of 0.989 and a root mean squared error of 1.683 in the test set. These results show the suitability of the machine learning tools combined with spectroscopic data as a screening method for the quality control of fruit juices. In addition, a prototype application has been developed to share the models with other users and facilitate the detection and quantification of adulteration in juices.

## 1. Introduction

Food adulteration is a fraudulent practice, carried out for mainly economic reasons. [1]. This illegal practice is one of the most important issues facing the agri-food industry nowadays since, in addition to being a consumer deception and an economic fraud, it can seriously damage the health of consumers who may suffer allergic reactions as they are unaware of the real content [2]. Numerous products are susceptible to these adulterations, which are becoming more and more sophisticated in order not to be detected, and one of the most frequent is fruit juices [3]. In addition, in Europe there is a strict regulation to guarantee the quality of juice production, regulated by the 2012/12 EU directive [4].

In the beverage industry, one of the most important sectors is juice production, and according to “Fruit Juice Market: Global Industry Trends, Share, Size, Growth, Opportunity and Forecast 2021–2026”, the fruit juice market reached a volume of 44.12 billion liters in 2020 and is expected to grow steadily in the coming years [5]. Generally, fruit juices are adulterated by adding sugars, by diluting them with water, or by mixing them with cheaper fruit juices. This illegal practice is carried out by some manufacturers to satisfy the high demand for the product, to hide low-quality raw materials, and to obtain higher economic benefits [6]. Juice-to-juice adulterations are very popular because they are complex matrices that are difficult to detect, which makes them very sophisticated [7], and grape juice is one of the most used for adulteration due to its low cost [8].

Therefore, the quick identification of this type of fraudulent practice is very necessary for consumers and regulatory agencies. For this reason, it is urgent for the food industry to have suitable analytical techniques to identify the different juice adulterations. Thus, different methodologies have been proposed, such as nuclear magnetic resonance [9,10], DNA-based techniques [11,12], and inductively coupled plasma mass spectrometry (ICP-MS) [13,14], among others. The most widely analytical techniques used for this purpose have been both liquid and gas chromatography [15,16,17,18]. However, all these techniques have limitations such as complexity, high cost, long analysis times, poor portability, and the need for a qualified operator. Therefore, it is convenient to provide other techniques without these limitations. In this sense, spectroscopy stands out as being the most used Fourier transform infrared spectroscopy (FT-IR). This has been used for the detection of sugars in different juices [19,20,21,22] as well as for juice-to-juice adulteration and authentication [23,24,25]. Another technique that has given excellent results but has been employed less than FT-IR is near-infrared spectroscopy (NIR). This technique has previously been used to detect the addition of sugars in apple juice [21] and bayberry juice [26]. Additionally, it has been successfully used to determine the quality of juice from different fruits [27], as well as to determine the addition of water in bayberry juice [28] and for the detection of adulterations in lime juice [29]. However, to the best of the authors’ knowledge, this technique has not been previously used to detect and quantify juice-to-juice adulterations. This is probably because the detection and quantification of this type of adulteration based on specific NIR signals are very complex due to the similarity of the adulterated and non-adulterated samples. Therefore, a different approach would be much more interesting, specifically using the NIR data as the dataset from a multisensory device, then not using some parts of the chemical information contained in the data but only the information needed for classification purposes.

Spectroscopic techniques are very suitable since they allow the analysis of the sample in situ as they are highly portable. In addition, they have numerous advantages over other traditional methods such as speed of analysis (less than 1 min), low sample preparation, reduced cost, and ease of use. Furthermore, NIR is a non-destructive technique, which offers information about the characteristics of the sample that are closely related to the chemical bonds present in them [30]. This information provides a spectralprint characteristic of the internal components of each sample. However, the analyst often only focuses on the identification of a few target signals to discriminate samples, even though it is possible to use all or almost all the signals recorded as a spectralprint. In these cases, a larger amount of information is obtained that is more complicated to manage. However, in combination with machine learning (ML) tools, it can be used to create models for sample characterization [31]. In this way, the use of ML tools and spectralprints for processing the data avoids the individual identification of target compounds, and this allows us to automate the process, reduce time, and eliminate subjectivity. It is noteworthy that juices are complex matrices, so determining target compounds can be a difficult and, in some cases, almost impossible task. Therefore, using all the information facilitates the authentication of the product because it takes into account a multitude of small differences that may go unnoticed in the individual identification of target compounds and yet may be of interest.

ML tools have been previously used in combination with the NIR spectrum for the rapid detection of authenticity in different foods including honey [32,33], meat [34], and milk [35,36]. 

Nowadays, classification and regression ML models in combination with spectroscopic data are becoming more and more popular. In the case of classification problems, the most used is the linear discriminant analysis (LDA), and in regression problems, it is the partial least squares (PLS) method [37,38]. Both models are linear and have excellent results, especially when there is a linear relationship between the spectrum and the variable to be determined. However, in situations where there is no linear separation, these models are not optimal. In these cases, the use of other models such as support vector machines (SVM) or random forest (RF) is required, which are being used more and more and offer excellent results [39]. Furthermore, in the context of juice analysis, a recent study obtained better results using SVM models than using LDA on data from an electronic nose system [40]. Another study provided better results with the RF model versus LDA, PLS, or SVM in the characterization of strawberry juice using data from an electronic nose and tongue system [41]. However, the literature on the authenticity of juices with such models using NIR is scarce.

Therefore, this study aims to develop a screening method based on NIR spectroscopy, for the detection of juice-to-juice adulterations. For this purpose, different types of juices (orange, apple, and pineapple) were adulterated with grape juice at different percentages. Both the most common linear models (PLS and LDA) and nonlinear models based on SVM and RF were evaluated.

## 2. Materials and Methods

### 2.1. Samples

Three types of fruit juices (pineapple, apple, and orange) were chosen, and grape juice was used as an adulterant due to its lower cost. For each of them, three different brands were selected and three batches of each, except for orange, where four different brands were used (one with two batches, and three with three batches). All the samples were purchased from local markets. The samples were labeled first with the abbreviation of the type of juice used: “O” for orange, “P” for pineapple, “A” for apple, and “G” for grape, followed by the batch and brand used. Finally, the samples were analyzed in duplicate so that an orange sample of the “HC” brand from the third batch would be labeled “O3_HC_R1” for the first replica and “O3_HC_R2” for the second replica. The total number of samples analyzed was 76 with a minimum of 18 samples for each type of juice.

### 2.2. Adulteration

To prepare the adulterated samples, firstly, two additional new samples were prepared from each type of juice, which consisted of an equal mix of all the juices from different brands, randomly selecting the batch. Each new additional sample of pineapple, apple, and orange juice was adulterated with the new additional sample of grape juice at different proportions. This procedure was carried out to ensure the greatest heterogeneity possible in the adulteration process. The adulteration levels prepared were: 5%, 10%, 15%, 20%, 30%, 40%, and 50% since they are the most common in food adulterations [42]. In addition, each percentage was analyzed in duplicate; therefore, the total number of samples was 108: 2 additional different samples × 3 types of juices × 9 adulteration percentages (including 0% and 100%) × 2 replicates. Samples were labeled first with the juice abbreviation (“O”, “P”, “A”) together with the additional sample used (1 or 2), followed by the percentage of adulteration (0, 5, 10, 15, 20, 30, 40 and 50) and finally “R1” or “R2” for the first or second replicate. Thus, the first replicate of a 10% adulterated orange juice from the first sample would be labeled as “O1_10_R1”.

### 2.3. Near-Infrared Spectroscopy (NIR)

The FOSS XDS Rapid Content™ Spectrometer (FOSS Analytical, Hilleroed, Denmark) was used for sample analysis. This equipment has a single light beam analyzer, and the spectra were acquired from 400 to 2500 nm with a resolution of 0.5 nm. Since the samples are liquid, it was necessary to use a cuvette with a gold reflector, using a 0.5 cm pathlength. Distilled water was used as a blank. For each sample, two spectra were scanned, and the final result was the average of both. The total analysis time per sample was 30 s.

### 2.4. Data Analysis

The spectrum of each of the samples was obtained and placed in matrices D_nxp,_ where *n* refers to the number of samples and *p* to the number of variables (wavenumber), i.e., the final complete array was D_184×4200_. Data analysis was carried out using RStudio software version 4.0.2 (RStudio Team 2021, Boston, MA, USA), using different packages. These include *ggplot2* [43] and *factoextra* [44] used for graphical representations, the *prospectr* package [45] to apply the first derivative and the Savitzky–Golay filter, the *mclust* package [46] for carrying out model-based clustering, *caret* [47] for the application of the different algorithms for both classification and regression, and *shiny* [48] for the development of the application.

## 3. Results and Discussion

### 3.1. Unsupervised Analysis

Unsupervised analyses are algorithms based on the training process on a data set without previously defined labels or classes, and this part of the study aimed to observe if there were differences in the spectra of each type of juice analyzed. For this reason, the data matrix for the unsupervised analysis consisted of the 76 unadulterated juice samples and the 4200 variables (wavenumbers), that is, D_76x4200_.

First, to improve the spectral resolution and compensate for baseline shifts and light scattering differences, the original spectra must be pretreated before further data analysis [49]. In this case, the first derivative was calculated to eliminate the problem of overlapping peaks and baseline shifts. In addition, a Savitzky–Golay smoothing filter (polynomial degree 3 and window size 11) was applied to reduce random noise. It should be noted that by applying this filter the information of the edges is eliminated and thus the number of variables was also reduced to 4190 corresponding to 402.5 nm to 2497.0 nm. Therefore, the resulting data matrix was reduced to D_76×41__90_.

**Figure 1:**
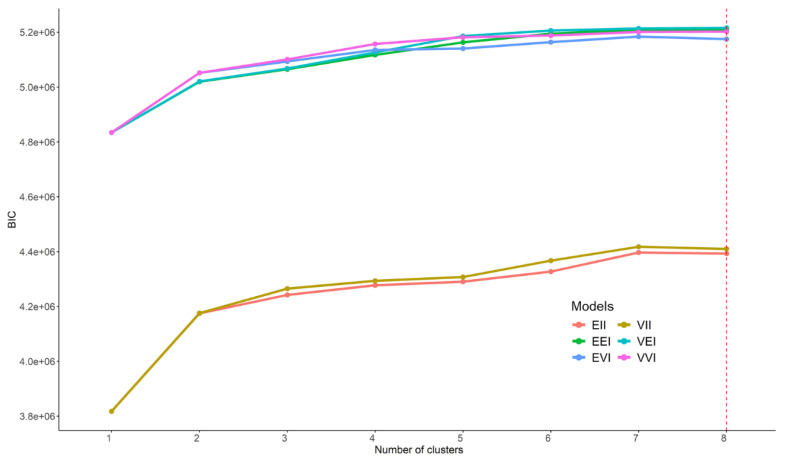
BIC value obtained as a function of the number of clusters per Gaussian mixture model resulting from the model-based clustering analysis. The spectroscopic data matrix of the unadulterated samples was used for the analysis, i.e., D_76×4190_.

To evaluate the grouping tendency of the samples, all the unadulterated fruit juice samples were subjected to Model-based clustering, specifically using Gaussian mixture models, which is an unsupervised technique that allows patterns in the data to be found. In this case, the number of Gaussian distributions was evaluated, with all the possible combinations of volume, shape, and orientation of each cluster that is determined by the covariance matrix. Since the number of samples is less than the number of variables, the different options evaluated are those corresponding to the spherical and diagonal distributions, which are the following: EII, VII, EEI, VEI, EVI, and VVI, where E indicates equal, V variable and I coordinate to the axes. In addition, the first identifier refers to volume, the second to shape, and the third to orientation. All this information and abbreviations have been previously described by the authors of the Mclust package [46]. The first two combinations (EII and VII) belong to the spherical Gaussian distribution since the shape is coordinated to the axes (identifier I in the second position) and, being a sphere, it makes no sense to consider the orientation of the axes. The rest of the combinations belong to diagonal distribution (EEI, VEI, EVI, and VVI) because they have the same orientation according to the axes; therefore, the identifier I appears in the last position. The best of the models was chosen based on the Bayesian information criterion (BIC); therefore, in this case, the higher the value, the better the evidence in favor of the model. Figure 1 represents the BIC value (*y*-axis) as a function of the number of clusters (*x*-axis) for each of the models (represented with colors).

As can be seen, in general, the higher the number of clusters, the higher the value of the BIC, regardless of the model used. It is also observed that the best result is obtained with eight clusters with the VEI model (maximum number of partitions tested); however, after four clusters, the increase in the BIC value is very subtle. The best model obtained with four partitions is the VVI, a Gaussian diagonal distribution, where the volume and shape are variable in each cluster and the orientation is coordinated to the axes. In this case, four groups must be obtained, one for each type of juice used (orange, pineapple, apple, and grape); for this reason, it was decided to adjust the model-based clustering with four groups and use the VVI distribution. In addition, as mentioned above, the increase in the BIC value is not very remarkable from four to eight clusters, so the model with four groups reflects the distribution of the data correctly. 

**Figure 2:**
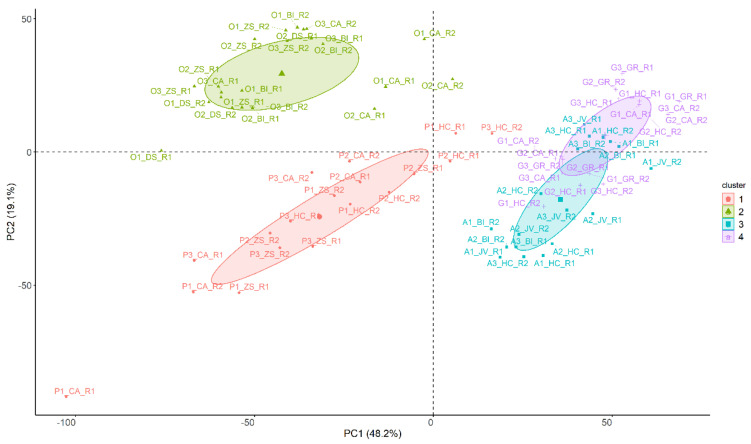
Representation of the samples as a function of the first two components using spectroscopic data matrix D_76×4190_. The samples have been colored and symbolized according to the group obtained by the model-based clustering (VVI distribution), where the centroid of each group is represented by the respective larger symbol and its distribution is shown as an ellipse.

To evaluate the distribution of the samples in the space, a principal component analysis (PCA) was performed by using the same data matrix (D_76×4190_). In Figure 2, the samples were represented according to the first two principal components (PC1 and PC2) which represent 48.2% and 19.1% of the variability of the data, respectively. The samples were colored based on the group obtained by the previously selected clustering method (model-based clustering with four groups and VVI distribution). In addition, this figure also shows the group assigned to each cluster as well as its distribution and centroid.

As can be seen in Figure 2, focusing on the information provided only by PCA (PC1 and PC2 explain 48.3% and 19.1% of the data variability, respectively), solely most of the orange juice samples can be easily differentiated from the rest, obtaining negative values for PC1 and positive values for PC2. In addition, a small difference is observed within this group depending on the brand where “CA” samples are located further away from the rest. The grape and apple juice samples do not seem to differ from each other, since most of the samples from both groups are overlapped as they present similar values for both PCs. In addition, some samples of pineapple juices (three of the six samples of the “HC” brand) appear in this location. However, the rest of the pineapple juice samples seem to differ in PCA, obtaining negative values for PC1 and PC2. 

Therefore, this analysis shows a grouping trend based on the type of fruit juice, where the brand seems to have a slightly minor influence on the spectrum, and the batch used does not seem to affect it. However, it does not provide perfect separation of the samples based on the raw material. It is important to point out that the adulterant (grape juice) is not distinguished from the rest so it would be complicated for its detection in a case of real adulteration. 

Moreover, in Figure 2 it is observed that the groups made by the model-based clustering are different from the information obtained by the PCA. In this case, although the grape juice samples appear mixed with the rest, the model can group them into a single group (colored purple). This is especially important given that grape juice is used for adulteration; therefore, its correct differentiation facilitates the detection of these fraudulent practices. It is also observed that each of the other three groups formed by the model-based clustering contain exclusively the samples corresponding to one type of juice. Thus, the orange juice samples are in the green cluster, the pineapple juice samples are in the red cluster and the apple juice samples are in the blue cluster. Finally, since the fitted model is VVI, it is observed that the clusters have a diagonal distribution where the volume and shape are variable, with orientation coordinated to the axes. In addition, the centroids of each of the four groups are represented with a respective symbol of a larger size.

This analysis showed a tendency to classify the juices according to the type of fruit, and to a lesser extent, there is an influence according to the brand used and a similar trend has been observed in a previous study using data from FT-IR analysis [25]. Model-based clustering has allowed the classification of grape juice samples in an accurate way within the same group, separating them from the rest of the samples. This trend is very useful in the detection of adulteration, and it was not possible to observe it using PCA. However, as discussed above, this model is a non-supervised technique where the information corresponding to the label (type of fruit juice) is not provided, so it cannot be used to predict future observations. For this reason, and once it has been verified that there is a classification trend, a supervised analysis must be used to detect and quantify future adulterated samples.

### 3.2. Supervised Analysis

#### 3.2.1. Classification Models

In supervised analysis, algorithms are trained on a set of previously labeled data, and the purpose of this part of the study was to create classification models to detect the adulterant (grape juice) in the different types of juices (orange, pineapple, and apple). Therefore, the complete data matrix which includes adulterated and non-adulterated samples was used (D_184×4190_-with first derivative and Savitzky–Golay filter) and four groups were established a priori based on the fruit juice: “orange”, “apple”, “pineapple”, and “adulterated”. The “adulterated” group contains the different types of pure juices (apple, pineapple, and orange), specifically adulterated using different grape juice percentages (5%, 10%, 15%, 20%, 30%, 40%, and 50%). 

The trained models include both parametric techniques such as linear discriminant analysis (LDA) as well as non-parametric techniques such as random forest (RF) and support vector machine (SVM). Before developing the models, the complete data set (D_184×4190_) was divided into two subsets, one containing 80% of the observations which constitute the training set to create the models, and the remaining 20% that constitutes the test set used to evaluate the performance of the models generated. Both subsets have been chosen in a balanced way to ensure that samples of all percentages and juices are present in each subset. Table 1 summarizes the accuracy obtained for each of the models in both the training and test sets.

##### Linear Discriminant Analysis (LDA)

In the LDA analysis, no hyperparameters need to be fitted, so the model was developed directly with the training set. In the case of the training set, the accuracy was 100%, and in the test set, it was 97.67%. Thus, only one unadulterated apple sample was predicted as adulterated. In addition, the kappa statistic was 0.9648, which considers the probability that a prediction is correct simply by chance. In general, it is established that a kappa value between 0.8 and 1 results in excellent model performance [46].

##### Support Vector Machine (SVM) with Gaussian Kernel

The SVM with a Gaussian kernel contains two hyperparameters that must be adjusted. One is called cost (*C*) and controls the penalty for misclassified observations and the other is called gamma (*γ*) which controls the flexibility of the model. Both must be chosen previously to control the balance between bias and variance of the model. For this purpose, an exponential growth search method was used, as described in previous studies [41,50,51]. This consists of taking values in the range of −10 to 10 for log_2_*C* and log_2_*γ,* and the best combination of the hyperparameters is the one that achieves the highest accuracy value for a 5-fold cross-validation on the training set itself. The accuracy for each combination of hyperparameters is represented in Figure S1 (supplementary material). In this case, the best value was obtained for a *C* of 2 and *γ* of 9.766 × 10^−4^. With these values, 100% accuracy was obtained in the training set and 88.37% in the test set. In this case, the kappa value was 0.8139. Although this value is considered good performance, it is still lower than the one obtained with the LDA model.

##### Random Forest (RF)

In the RF models, there are two hyperparameters to be adjusted. The first one is the number of trees, which was kept at 500 since the error was stabilized with this value. The second is the number of predictors evaluated before each division (*mtry*) which acquired the value of 65 since for classification problems it is recommended to use the square root of the total number of predictors [52]. This combination of values provided an accuracy of 100% for the training set and 97.67% for the test set. In this case, the same sample as in the LDA was misclassified (A3_JV_R2) which was predicted as adulterated. Therefore, the value of the kappa statistic was 0.9648. This performance was the same as that obtained with the LDA. In addition, once again, a sample of unadulterated apple juice was predicted and misclassified as adulterated. This could be because the spectra of apple and grape juices are more alike, and the information provided by the PCA (See Figure 2—Exploratory analysis) placed these two types of fruit as the same.

To sum up, the best performances were obtained with LDA and RF models while SVM reported worse results. Therefore, no difference has been found between the parametric (LDA) and non-parametric (RF) methods. However, a previous study reported better results in detecting adulteration using SVM models instead of LDA [40], and others reported similar results [25,41].

#### 3.2.2. Regression Models

Once the models to detect the presence of adulterant (grape juice) in the different juices (orange, apple, and pineapple) have been developed, the next step was to evaluate the suitability of the technique to quantify the percentage of adulteration. For this purpose, a global regression was performed using all the samples generated in the adulteration process. Therefore, the matrix is constituted by 96 samples (D_96×4190_-with first derivative and Savitzky–Golay filter) which were divided again into two subsets: (I) a training set, which is made up of 80% of the samples and used to create the regression models, and (II) a test set, which consists of the remaining 20% of the samples and is used to evaluate the performance of the models. The splitting was done in a balanced way: the test set contains at least one sample of each type of juice and the percentage of adulteration. Before the creation of the regression algorithms, pretreatment of the data was necessary, which consists of selecting variables by applying the Boruta algorithm to the training set. This selection made it possible to reduce the number of variables from 4190 to 95, which are related to the response variable (percentage of adulteration). Specifically, this algorithm identified 95 significant variables for the prediction of adulteration, 33 tentatives, and 4062 rejections. Once the significant variables were selected, different regression models were created with the training set. Both non-parametric (SVR and RF regression) and parametric (PLS) models are included. A summary of the main performance statistics of the different models is shown in Table 2.

##### Partial Least Squares Regression (PLS)

The optimal number of components (from 1 to 15) was evaluated by leaving one out cross-validation (LOOCV) on the training set. The evolution of the root mean squared error RMSE as a function of the number of components is shown in Figure S2 (supplementary material), and the best result was obtained with 11 components as it allowed the greatest reduction in RMSE. The final model led to an R^2^ of 0.951 and RMSE of 3.644 for the training set and an R^2^ of 0.931 and RMSE of 4.388 for the test set. The high R^2^ indicates a strong correlation of the wavenumber with the response variable but the RMSE is high to accurately predict the percentage of adulteration.

##### Support Vector Regression (SVR)

The optimization method for the hyperparameters was the same as in the classification SVM model. In this case, the hyperparameter ε which controls the learning rate of the model was kept constant at 0.1. The RMSE for each combination of hyperparameters is represented in Figure S3 (supplementary material). The best values of *C* and *γ* were 22.63 (log_2_*C* = 4.5) and 5.52 × 10^−3^ (log_2_*γ* = −7.5), respectively. The new model obtained an R^2^ of 0.994 and 0.989 for the training and test set, respectively, while the RSME was 1.446 and 1.683. This model allows us to predict the percentage of adulteration in a very significant way.

##### Random Forest Regression

The number of trees was kept constant at 500 since the error is stabilized at this value. However, the value of *mtry* was optimized by LOOCV on the training set by testing values ranging from 1 to 50. The evolution of the RMSE for each of the values of *mtry* tested is shown in Figure S4 (Supplementary Material) and the one that achieved the greatest reduction in RMSE was 6. This new model led to an R^2^ of 0.983 and 0.851 and an RMSE of 2.571 and 7.223 for the training and test set, respectively.

These results are worse than those obtained with PLS and SVR. In addition, there is overfitting in the training set, and therefore, the R^2^ decreases and the RMSE increases significantly in the test set. In this case, it could be considered that the statistics of the parametric PLS technique and the non-parametric RF technique are not satisfactory enough for the quantification of the adulterant. However, the non-parametric technique (SVR) obtains significant results and allows us to predict the percentage of adulteration accurately. It should be noted that a previous study also obtained better results using SVR models for the detection of adulterants in fruit juices [25].

### 3.3. Application Development 

The spectra in combination with ML tools have obtained excellent results for the detection and quantification of adulterants in juices. For this reason, a simple web application has been created to share the previously trained RF model for detection and the SVR model for quantification. It should be noted that the algorithms are not usually shared, which makes monitoring of the analyzed samples difficult for regulatory agencies and other users. The availability of such models prevents the user from having their own database, in addition to saving time and effort in the characterization of the samples. Another great advantage is that any user without previous knowledge of ML tools could use the models, which can be found in the following link: https://joseluispecalle.shinyapps.io/Adulteration_Juices_App/. (Accessed on 28 May 2021).

To use this application, it is only necessary to upload the excel or csv file generated from the analysis of the sample by NIR. In the “Download” button, a test file has been inserted, which can be used to check the application functionality. Once the file is uploaded and the “submit” button is clicked, the application will directly perform the first derivative and the Savitsky–Golay filter to predict if the juice is adulterated using the previously trained RF model. If adulteration is detected, it automatically performs the variable selection by the Boruta algorithm and quantifies the percentage of adulteration using the previously trained SVR model. 

Finally, it should be noted that the application can be improved with the introduction of new functionalities. In addition, like the human taste system, these algorithms can “learn” as they are “taught”, i.e., more and more samples will be analyzed, thus getting closer to reality and satisfying the demands of today’s beverage industry.

## 4. Conclusions

The dataset from the infrared spectroscopic analyses has been used for the detection of the adulteration of several fruit juices with grape juice. The spectroscopic data in combination with model-based clustering has made it possible to visualize a clustering trend depending on the type of fruit juice (orange, pineapple, and apple). In addition, the use of supervised ML techniques has allowed us to detect the adulterant (with an accuracy of 97.67% for the LDA and RF models) and to quantify the percentage of adulteration (R^2^ higher than 0.98 and RMSE lower than 1.7 for the SVR model). In addition, the availability of a methodology based on spectroscopic data allows us to obtain a faster, more objective, and cheaper result than with traditional methods. Like the human taste system, the developed method can be upgraded as it learns when it is trained with more samples. Furthermore, it is a non-destructive technique with high portability which could be used for routine in situ control analysis of fruit juices by regulatory agencies and industries.

## Figures and Tables

**Table 1:** Accuracy and kappa results for the different classification algorithms applied on the complete spectroscopic data matrix (D_184×4190_).

	Employed Value	Training Set		Test Set	
Model	Hyperparameters	Accuracy (%)	Kappa	Accuracy (%)	Kappa
**LDA**	-	100	1	97.67	0.9648
**SVM**	*C* = 2 *γ* = 9.766 × 10^−4^	100	1	88.37	0.8139
**RF**	*mtry* = 65 Number of trees = 500	100	1	97.67	0.9648

**Table 2:** Results obtained for each regression method applied in the quantification of the global adulterant by using the spectroscopic data matrix of all adulterated juice samples (D_96×89_).

Model	Hyperparameter	Training Set Performance	Test Set Performance
**PLS**	11 principal components	RMSE = 3.644 R^2^ = 0.951	RMSE = 4.388 R^2^ = 0.931
**SVR**	*C* = 22.63 *Y* = 5.52 × 10^−3^	RMSE = 1.446 R^2^ = 0.994	RMSE = 1.683 R^2^ = 0.989
**RF**	*mtry* = 6 Number of trees = 500	RMSE = 2.571 R^2^ = 0.983	RMSE = 7.223 R^2^ = 0.851

## Data Availability

Not applicable.

## Supplementary Materials

The following supporting information can be downloaded at: https://www.mdpi.com/article/10.3390/s22103852/s1, Figure S1: Search for the best combination of hyperparameters (C and γ) for the Gaussian SVM model for adulterant detection. The result is obtained by the CV of 5 folds using the spectroscopic data matrix of all training set samples (D_138x4190_); Figure S2: Evolution of the root mean square error (RMSE), as a function of the number of components used in PLS analysis. The LOOCV error has been used for the spectroscopic data matrix of the adulteration process samples from the training set (D_72x89_); Figure S3: Search for the best combination of hyperparameters (C and γ) for the Gaussian SVM model for adulterant detection based on the RMSE. The result is obtained by LOOCV using the spectroscopic data matrix of the adulteration process samples from the training set (D_72x89_); Figure S4: Evolution of the root mean square error (RMSE), as a function of the mtry value used in RF analysis. The LOOCV error has been used for the spectroscopic data matrix of the adulteration process samples from the training set (D_72x89_).
