# Supplementary figures and images for: Rapid Detection and Quantification of Adulterants in Fruit Juices Using Machine Learning Tools and Spectroscopy Data
Source: Sensors (Basel). 2022 May 19;22(10):3852. doi: 10.3390/s22103852 (PMC9145498; doi:10.3390/s22103852)

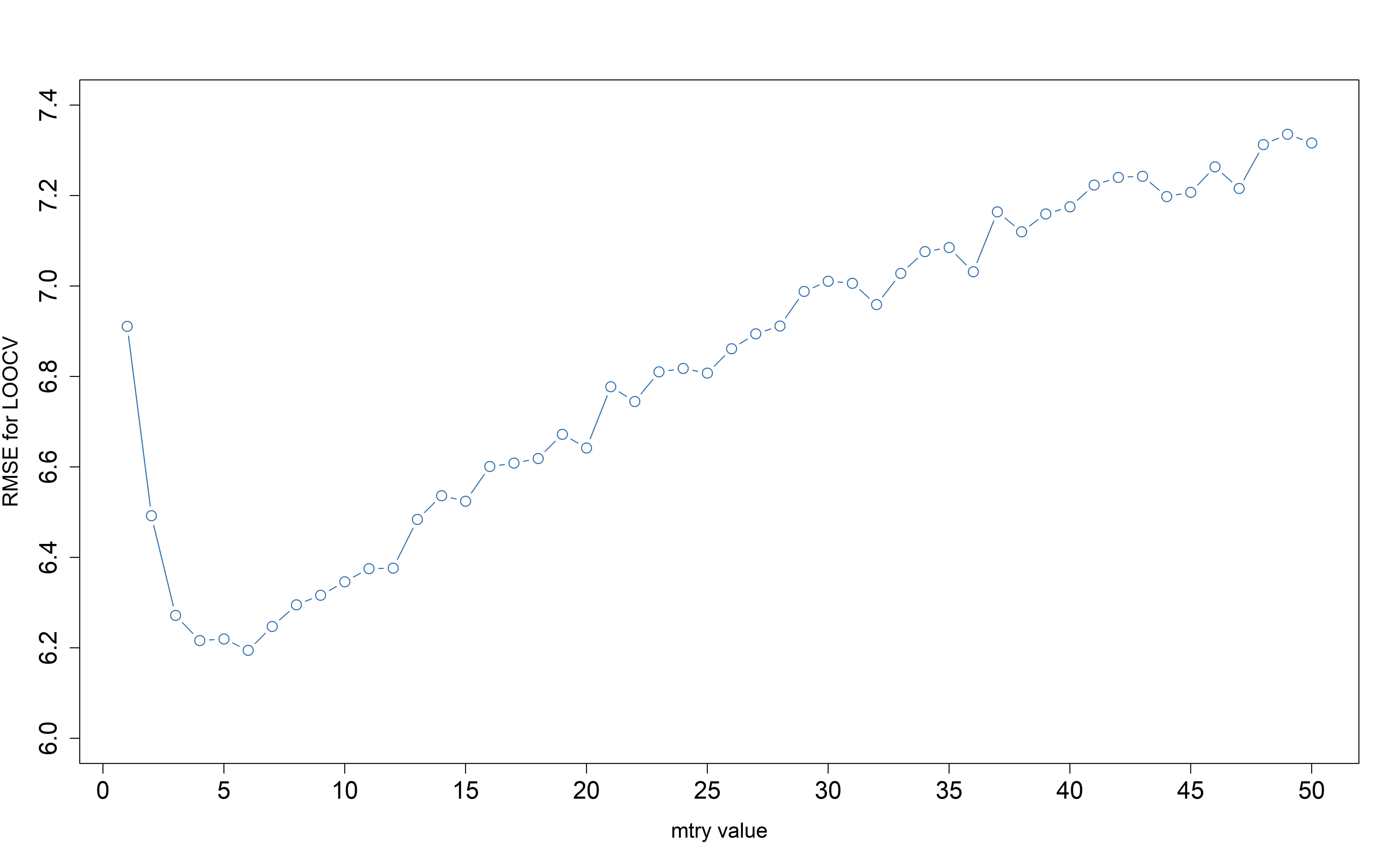

Supplement: Supplementary file 1 [file sensors-22-03852-s001.zip › Figure S4.png]
